# Optimization and Control of Agent-Based Models in Biology: A Perspective

**DOI:** 10.1007/s11538-016-0225-6

**Published:** 2016-11-08

**Authors:** G. An, B. G. Fitzpatrick, S. Christley, P. Federico, A. Kanarek, R. Miller Neilan, M. Oremland, R. Salinas, R. Laubenbacher, S. Lenhart

**Affiliations:** 1 Department of Surgery, University of Chicago, Chicago, IL USA; 2 Department of Mathematics, Loyola Marymount University, and Tempest Technologies, Los Angeles, CA USA; 3 Department of Clinical Science, University of Texas, Southwestern Medical Center, Dallas, TX USA; 4 Department of Mathematics, Computer Science, and Physics, Capital University, Columbus, OH USA; 5 U.S. Environmental Protection Agency, Washington, DC USA; 6 Department of Mathematics and Computer Science, Duquesne University, Pittsburgh, PA USA; 7 Mathematical Biosciences Institute, Ohio State University, Columbus, OH USA; 8 Department of Mathematical Sciences, Appalachian State University, Boone, NC USA; 9 Center for Quantitative Medicine, UConn Health, and Jackson Laboratory for Genomic Medicine, Farmington, CT USA; 10 Department of Mathematics and NIMBioS, University of Tennessee, Knoxville, TN USA

**Keywords:** Agent-based modeling, Systems theory, Optimization, Optimal control

## Abstract

Agent-based models (ABMs) have become an increasingly important mode of inquiry for the life sciences. They are particularly valuable for systems that are not understood well enough to build an equation-based model. These advantages, however, are counterbalanced by the difficulty of analyzing and using ABMs, due to the lack of the type of mathematical tools available for more traditional models, which leaves simulation as the primary approach. As models become large, simulation becomes challenging. This paper proposes a novel approach to two mathematical aspects of ABMs, optimization and control, and it presents a few first steps outlining how one might carry out this approach. Rather than viewing the ABM as a model, it is to be viewed as a surrogate for the actual system. For a given optimization or control problem (which may change over time), the surrogate system is modeled instead, using data from the ABM and a modeling framework for which ready-made mathematical tools exist, such as differential equations, or for which control strategies can explored more easily. Once the optimization problem is solved for the model of the surrogate, it is then lifted to the surrogate and tested. The final step is to lift the optimization solution from the surrogate system to the actual system. This program is illustrated with published work, using two relatively simple ABMs as a demonstration, Sugarscape and a consumer-resource ABM. Specific techniques discussed include dimension reduction and approximation of an ABM by difference equations as well systems of PDEs, related to certain specific control objectives. This demonstration illustrates the very challenging mathematical problems that need to be solved before this approach can be realistically applied to complex and large ABMs, current and future. The paper outlines a research program to address them.

## Introduction

Technological advances in data generation and in computer hardware and software have transformed the life sciences from being data-poor to being data-rich. Computation is now an essential component of much research in biology, and it is also becoming ubiquitous across biomedicine and healthcare. As in engineering and science generally, a great deal of recent progress in the life sciences now relies on computation, which has come to be recognized as a “third pillar of science,” together with theory and experimentation (Smarr 1992; President’s Information Technology Advisory Committee 2005). At a fundamental level, such computational models are constructed for two distinct but often entangled purposes: (1) models to increase understanding of the system being modeled, and (2) models to inform decisions to be made about the system being modeled. While clearly these goals overlap and can be thought of as generally existing on a continuum from understanding to decision support, and there are varying and domain-specific criteria for the trustworthiness of that transition, in applied sciences, such as biomedicine, there is a desire to develop investigatory pathways to move toward using modeling and simulation as a means of engineering control strategies. This process involves the translation of methods and concepts that have been demonstrated to be useful in other domains. For instance, many problems in the life sciences can be viewed from the point of view of optimal control and optimization, an area to which the mathematical sciences have made substantial contributions through mathematical modeling and algorithms. Yet, mathematical approaches to analysis have not been directly applicable to a type of model that has gained in popularity across the life sciences in recent years: agent-based models (ABMs). ABMs are characterized by their ease of construction by domain experts, ability to capture spatial heterogeneity, and faithful representation of local characteristics that generate global dynamics. The principal method of analysis for ABMs remains extensive simulation. As these models grow larger and more complex, even simulation quickly reaches computational limits. The purpose of this article is to discuss how mathematical approximations of ABMs could be developed, in particular for optimization and control purposes, in order to overcome these limitations. We offer the following rationale for our approach:

1. We consider ABMs “middle-ware” investigatory objects: selectively abstracted representations of the real-world system that are yet too complex for traditional formal mathematical analysis. To a great degree this complexity arises out of system properties that resist traditional modeling methods, such as variable component-component interactions, spatial heterogeneity and insufficiency of mean-field approximations, and this makes ABMs “sufficiently complex” proxies for the real-world system.
2. The fact that ABMs are computational constructs vastly increases the range of “experimental” conditions able to be applied versus their real-world referent (or real-world physical proxy models); this includes testing putative control strategies.
3. However, comprehensive search of model-response space using brute force embarrassingly parallel simulation is computationally expensive, and may not be necessary. Therefore, identifying methodological bridges between ABMs and more formally tractable SLMs would be beneficial and serve two purposes:
  - To reduce the search space for putative controls, which can then be tested via more tractable embarrassingly parallel simulation experiments; and
  - To facilitate iterative refinement/expansion/reduction of an existing ABM in reference to its intended use with respect to its referent (in this case, the search for practically implementable control strategies).
4. By virtue of being “sufficiently complex” proxy systems, information and knowledge obtained by examining ABMs subject to control *may* provide insight into how to effectively control the real-world referent. At the very least, this process can provide a first approximation of the set of putative controls.

This rationale leads us to the following:

**Main hypothesis **If an ABM is treated not as a model of a system of interest but as the system itself, then simpler mathematical models can be derived that capture key features of the ABM for a particular control or optimization objective and, by extension, the biological system of interest.

One might argue that if there is an equation-based model that can be used to solve optimization problems related to the biological system, then one should have constructed such a model in the first place, rather than build an ABM as an intermediary step. This might well be the right approach, if feasible, but there are several reasons why one might nevertheless want to build an ABM first. Firstly, of all model types, an ABM requires arguably the fewest simplifying assumptions to be made, and it can be validated in the most direct way, through, e.g., observation of characteristic patterns rather than surrogate summary statistics. If the biological system is not very well understood, this can be an important reason for an ABM as a first modeling step. Once an ABM is built and validated, it can be used to understand the system better, e.g., the importance of different variables or spatial features. Once a control objective is specified, this understanding can then lead to a possibly much simpler equation-based model that is faithful for the specific control objective, but possibly few or none of the other features. Secondly, the need for optimization and control might be an ongoing process, e.g., for models that are used for policy decisions, and the control and optimization objectives might change over time. In this case, the model is likely intended to capture all possible information about the system and incorporate new information as it becomes available. It is not efficient in this case to build a series of one-off *de novo* models for each. Thirdly, the model might be built by domain experts with little expertise in mathematical modeling, who can build an ABM with much greater ease than they can an equation-based model. Thus, the proposed indirect approach to optimization and control of systems is not intended to replace a direct modeling approach in all cases, but is intended to be used in cases where a direct approach is either not feasible or not desirable.

## Agent-Based Models

The life sciences frequently examine systems with interacting components at multiple levels of hierarchy and structure, from cells to connections between cells that lead to tissue-level properties, to the whole-organism level, and on to individuals in ecosystems. The reduction of biological systems to physical or chemical phenomena has yielded interesting insights at a fundamental level, but these approaches, to a great degree, fail to sufficiently represent the range and complexity of biological behavior that is often of interest at a level relevant for optimization and control approaches. Biological systems are distinguished by an organizational structure that generates multi-scale phenomena arising from the complex interactions between their physical components. They support adaptive behavior of individuals and exhibit great individual variability, whether at the scale of molecules or humans. The non-linearities associated with the functional transitions between organizational scales challenge the application of many traditional mathematical methods, particularly those oriented toward engineering means of controlling those systems. However, ABMs, which typically simulate interactions between individual components operating in heterogeneous spatial environments to generate population-level behaviors, often span two or even more organizational scales (e.g., molecular rules $<=>$ individual cell behavior $<=>$ cell population/tissue behavior $<=>$ multi-tissue/organism $<=>$ multi-organism/population). They have become an important technology for the life sciences because of their capacity to account for heterogeneity among components. Additionally, they are able to readily integrate knowledge with data because, in many cases, reductionist experimental data offer better observability of individuals than aggregates. Often, scientists find ABMs simpler to explain to stakeholders such as policy makers, in terms of components for which they have some intuition. It has now become relatively easy for a domain expert to construct an ABM, thanks to easy-to-use software interfaces for model construction, simulation, and visualization. For these and other reasons, ABMs have been increasingly adopted in the evolving area of computational simulation science. The notion that computation has come to complement experiment and theory as a third pillar of science arises from the use of complex computational simulations not only as a means of integrating and comparing theory and experiment but, more importantly, as tools to aid in theory construction, to illuminate crucial components and uncertainties, to generate and examine new hypotheses, to suggest new experiments and data collection efforts, and to strengthen policy development and decision-making.

The basic structure of an ABM consists of individuals/agents with attributes and rules of behavior, rules that govern agent actions and interactions with other agents, as well as the interaction of agents with a potentially complex heterogeneous environment. ABMs often allow for individual variation among agents, challenging the compartmentalization typically used in dynamical systems models. Moreover, agents may have adaptive behavioral rules that lead to unforeseeable interactions and emergence. The time scales of different behavioral rules and environmental pressures can also be quite variable. These features make ABMs difficult to encode in terms of traditional difference or differential equations models. The behaviors and rules of ABMs are typically encoded in software as simple logical rules, coupled with random number generation to model uncertain events and outcomes. As such, ABM construction is more accessible than mathematically involved approaches such as, e.g., systems of differential equations. This simplicity of implementation allows researchers to translate hypotheses into a computational form, so that the ABM plays the role of a digital “sand box,” aiding in the investigation and visualization, advancing theory through conceptual model falsification.

An important and primary feature of ABMs is the population-level aggregated behavior that emerges from the rule-based individual interactions, such as the patterns of segregation in the model of (Schelling 1971) or synchronization of breeding in birds (Railsback and Grimm 2012). While differential equations models like the Belousov-Zhabotinsky reaction model (see, e.g., Murray 2011) can exhibit similar complex pattern evolution, the diversity of pattern and structure formation in ABMs is remarkable (Epstein and Axtell 1996; Gilbert 2008; Railsback and Grimm 2012).

Scientific inquiry into the control points of a system and the key drivers of system behaviors, however, can be difficult with ABMs. For this purpose, computational objects such as cellular automata or modeling methods such as discrete event simulation can be thought of as special cases of ABMs, but to date, there is not currently a rigorous formal description of what constitutes an ABM. A major benefit of using ABMs is their ability to generate, through simulation, non-linear transitions between multiple scales of organization. However, determining parameter settings that lead to different patterns can be extremely difficult. In contrast, this is relatively straightforward for systems of differential equations, for example. Exhaustive simulation to investigate bifurcations and stability, though cheaper and faster than real-world experimentation, can be prohibitively expensive in terms of compute cycles and the resources needed to execute them. Very complex differential equations models may be similarly expensive to evaluate computationally, but the formal mathematical structure of systems of differential equation often permits analyses in a way that the interaction-based structure of an ABM does not. This difference accounts for the significant appeal of more traditional mathematical models. Reducing the complexity of a differential equations model for design and optimization studies can be challenging, but generally the route is clearer than it is with ABMs, features of which may frustrate attempts at reduction by formal inspection and model reduction methods.

A problem of practical interest is that of policy guidance. In distinction with basic scientific inquiry, ecological management, public health, and medical domains need tools for rational, evidence-based decision-making in treatments, interventions, and resource management problems. Models have been used successfully to support such efforts. Social and ecological applications often involve problems for which direct experimentation is, at best, difficult. For example, controlling non-native species such as the wild hog *Sus Scrofa* in the Great Smoky Mountains National Park (Peine and Farmer 1990) has created a number of political difficulties, and mathematical models are beginning to suggest management strategies (Salinas et al. 2015; Levy et al. 2016). As another example, college drinking is a major public health problem. Calls for a reduction in the minimum legal drinking age suggest the undertaking of a complex, large scale social and political experiment with potentially major consequences. Computational decision aids can support policy investigation when experimentation and testing must necessarily be limited (McCardell 2008; Fitzpatrick et al. 2012, 2016a). Again, exhaustive simulation of control strategies may not be a desirable or even viable option, and developing mathematically tractable tools for winnowing the vast array of control strategies or policies into a manageable set for simulation can enhance this type of model tremendously.

To illustrate just how widely applicable ABMs are, we point to several additional representative examples. At the population level, EpiSims (Eubank 2005; Stroud et al. 2007) is a very large population-level ABM that explicitly represents millions of individuals and their daily movements in a faithfully represented urban environment. This movement model is then overlaid with an epidemiological model that can be used to simulate the spread of a pathogen through the population. EpiSims has been used as a policy decision-making tool in several contexts. In Wang et al. (2014), an ABM is used to study the impact of social norms on obesity and eating behaviors among US school children. At the tissue scale in the human body, a wide variety of problems have been approached through ABMs. In Ziraldo and Solovyev (2015), an ABM is used for a computational study of treatment options for pressure ulcers in patients with spinal cord injuries. In Gong et al. (2015), an ABM of granuloma formation in tuberculosis is used as a platform for the *in silico* design of combination therapies with different antibiotics. The ABM is combined with a PDE model to accurately represent diffusion of different molecules through tissue. Many other examples can be found in the literature.

## Toward a Mathematical Approach to ABMs

There is a nearly irresistible pull, when developing an ABM, toward increasing levels of detail and complexity. The enormous flexibility of ABMs allows a modeler to create agents with many attributes operating in an environment that is heterogeneous in multiple dimensions and to build interaction rules that account for rich, complex behaviors and relationships. As the complexity grows, the dimensionality of the parameter space does as well, and the ability to conduct systematic inquiry becomes more challenging. The ABM structure that invites researchers with its developmental simplicity becomes a significant drawback: as noted above, the computational simulation based on logical rules and individual attributes can be quite resistant to formal mathematical analysis or even to experimental insights, if the model rules are too complex. One strategy for approaching these trade-offs is “pattern-oriented modeling (Grimm and Railsback 2005; Grimm et al. 2005), in which one balances model complexity against the ability of the model’s output patterns to match those observed in the real world (see also Thorngate and Edmonds 2013, for pattern analysis in ABMs). We suggest that analysis at a system level can benefit greatly from transformation of the ABM into mathematical formalisms that are more accessible to system-level analysis.

*As stated in our main hypothesis, we assert that ABMs can be used as proxy systems, rather than as the model to be analyzed directly, in an investigatory pathway that can lead to the development of control strategies for highly complex real-world systems.* The complexity of aggregate behaviors observed in ABMs, which is seen by many ABM modelers as unapproachable with system-level compartmental or aggregated models, offers new and exciting challenges to the systems theory community, calling for the creation of new approaches.

There are several examples in the literature that can be seen as first steps toward a research program of the kind we are advocating. In Roeder et al. (2006), an ABM is used to study the effects of treating chronic myeloid leukemia with the drug imatinib, known as Gleevec, a tyrosine kinase inhibitor that interrupts key signaling pathways in cancer cells, thereby inhibiting cell proliferation. While being very successful in achieving a substantial reduction in the number of malignant cells, this treatment rarely eliminates all such cells, leading to cancer recurrence. In Roeder et al. (2006), the model is used to provide evidence for a new hypothesis explaining lack of complete success, which implicates different effects of imatinib on malignant stem cells, leaving a residual pool that replenishes the repertoire of cancer cells after treatment ends. The model supports this hypothesis, which is also corroborated by patient data. The agents in the ABM are cells of different types. There is no explicit spatial environment; rather, cells are divided into two different environments, representing cell growth and quiescence. Cells can move between these environments, depending on different signals they receive. Imatinib treatment affects several different parameters in the model.

At each ABM time step, the model evaluates a collection of probabilistic rules that affects the state of each cell and its location in one or the other of the compartments. Due to the large number of rules to be evaluated, leading to significant computational cost, it is only possible to use a small fraction of the actual number of cells involved. Still, one simulation run of this large stochastic model requires on the order of 6 h, with hundreds of thousands of rules to be evaluated at each time step. In Kim et al. (2008), the authors developed a deterministic difference equations model, consisting of approximately 6000 equations, that faithfully reproduces the behavior of this ABM and can be simulated in a matter of seconds. Cells are clustered depending on their state, and there is no limit on the size of the clusters, so that the model can represent any number of cells. This clustering approach also allows the model to be deterministic rather than stochastic. Then, in Kim et al. (2008), a (deterministic) PDE model was presented that accomplished the same task, agreeing with both the ABM and the difference equations model in almost all aspects. The main difference is that continuous time causes some aspects of the model to behave differently from either of the discrete time models. We view this progression of models as a case study of how one can move from a complex and hard to execute ABM to a more easily manageable equation-based mathematical model that allows analysis.

## Optimization and Control

A well-known family of ABMs known as Sugarscape (Wilensky 2009; Epstein and Axtell 1996) has been used for the study of a variety of control processes in the life sciences, social science, and economics. The stochastic Sugarscape ABMs include agent heterogeneity, environmental heterogeneity, and accumulation of agent resources (i.e., sugar) over time, thus incorporating the main complexities frequently found in ABMs. Agents negotiate a spatial environment in search of a resource called sugar, with higher sugar concentrations represented as elevations in the landscape. Different agents have differing abilities to perceive sugar gradients, leading to different levels of agent fitness. Complete lack of sugar leads to agent death. Control is included as taxation of agents’ sugar resources, with the goal of maximizing a weighted combination of total taxes less a measure of the impact of taxation on the population. Recently, Christley et al. (2015) approximated a Sugarscape model using a system of parabolic PDEs. The goal was to explore optimal control scenarios for Sugarscape, applying mathematical optimization approaches to the PDE model. This approach performed well in scenarios in which the control was assumed to be constant. Optimal controls generated by applying optimal control theory to the PDE system provided time-varying tax rates specific to an agent’s location and current wealth. When implemented in the ABM, the optimal controls performed reasonably well even though some error was introduced between the PDE and ABM systems.

Several different approaches to Sugarscape control are described in Oremland and Laubenbacher (2014a, b), approaches which illustrate the philosophy advocated herein. One approach focuses on dimension reduction of the ABM (by reducing the number of spatial locations, agents, and other aspects), while preserving those model features relevant for a given control objective. This is done by applying a randomly chosen collection of controls to both the original and the reduced ABM and computing the similarity of the relative rankings of the controls for the two models. The user can then choose a level of similarity that is acceptable for a particular control objective, thereby deciding how closely the reduced model needs to fit the original one for the control purpose. Controls are then computed for the reduced model and lifted to the original one. Note that the reduced model might be dramatically different from the original one in other aspects. The advantage of the reduced model might be ease of computation, although care must be taken that this is indeed the case. The optimization method chosen is Pareto optimization. This multiobjective optimization method has the advantage that it computes a collection of controls, each of which has the property that optimality in one objective cannot be improved without losing optimality in another. Thus, it computes optimal control inputs for various weightings of the individual objectives. The user can then decide which to choose. Another approach that can be taken using either the original model, or, possibly more easily, the reduced model, is to approximate the ABM by an equation-based model that can be used for control. Again, the equation model might be adequate for a control objective without preserving many other important features of the ABM, e.g., spatial heterogeneity. In Oremland and Laubenbacher (2014b), for instance, the rabbits-and-grass model is approximated almost perfectly, for the purpose of rabbit control, by a pair of fairly simple difference equations. But these have phenomenological parameters and contain no information about the spatial aspects of the model. This is also illustrated with a Sugarscape example.

Another approach to approximation of ABMs and corresponding optimal control has been developed by Lenhart et al. (2015, 2016), using a system of stochastic partial differential equations with non-local terms as an approximation, with corresponding novel optimal control results. The control of this system is motivated by a model for optimal harvesting of a population on a spatial grassland habitat, which is described below.

*We interpret these examples as evidence that it is possible to approximate large, complex, stochastic ABMs with mathematical models that are easier to execute and can be analyzed with mathematical methods. *We will elaborate on this approach below, using a much simpler ABM as an illustrative proof of concept and further validation of our main hypothesis.

## A Case Study: Ecological Pest Control

To focus the discussion on mathematical issues, we consider a comparatively simple application problem in which an ABM is a natural and easily implemented model, and for which control policies are of interest. We consider a two-species consumer-resource structure in a two-dimensional spatial domain. The spatial domain is divided into discrete patches, and time progresses in discrete steps. The resource, which we call grass, is produced with a commercial goal in mind. The species, called rabbits, consumes the grass and hence degrades the commercial viability of the grass crop. The simple model we examine involves rabbits and grass distributed across a rectangular domain. This model is implemented in the NetLogo framework (Wilensky 1999, 2001) as “Rabbits–Grass–Weeds.” In this simple example, each rabbit’s state is characterized by four quantities: its lat-long position in space, the angle it faces, and its energy content. Energy content is measured by the amount of grass the rabbit consumes, and when the rabbit crosses an energy threshold, it produces one rabbit as offspring. Movement is governed by an angular random walk: the rabbit chooses two uniformly distributed angles (left, right), differences them, adds that to its current facing angle, and moves one unit in that direction. The grass state in each position patch is 0 or 1, denoting presence or absence. Grass grows from 0 to 1 at a random rate. Energy content of a rabbit corresponds to the number of grass patches consumed. If a rabbit’s energy level falls below a al threshold, the rabbit dies. There are a small number of parameters in the model: the grass growth probability, the rabbit’s angular field of vision (affecting its movement), as well as birth and death thresholds.

While we focus on this simple version of the model in this paper, the problem is easily made more complex in the presence of a few natural generalizations. First, the grass growth probability may be spatially heterogeneous, dependent on local soil and water conditions. Second, rabbits may be drawn to areas within the region of interest with the highest grass content, bringing about a directed random walk that is chemotactic in nature. Third, the birth and death probabilities of the rabbits may not be identical, potentially leading to “winners” and “losers” within the rabbit community. Even with these potential complexifiers, this model does lack some interesting and important properties that make ABM behavior so rich, such as agent adaptability. Nonetheless, this model does include stochastic variation among agents and modeling features (e.g., the nature of the agent movement and of the conversion of grass to rabbits) that are not easily treated by traditional SLM approaches. Furthermore, because the current goal of this paper is to demonstrate the mapping between ABMs and equation-based system-level models in order to apply optimal control methods, we have chosen the simplified version of the rabbit–grass ABM as the initial starting point for the investigation of creating the cross-platform mapping process. By using simplified models, we emphasize the mapping and connection between two different perspectives of representing knowledge about the system, i.e. individual-based knowledge versus aggregated system-level knowledge. Our stepwise approach will then attempt to perform this mapping with increasingly sophisticated ABMs and target system-level modeling methods (see below).

The available control is that of harvesting, implemented as a probability of being harvested. In each patch and at each time step, the harvesting probability is specified. A random number is generated, and, if that number is less than the harvest rate, any rabbits in the patch are harvested. In its simplest instantiation, this probability may be uniform across the region of interest, but one may also implement harvesting with greater effort in some areas than in others.

It is at this point that we see some difficulty in the agent-based formulation. One may attempt to “wrap an optimization loop around” the ABM in order to devise an optimal harvesting plan. However, the stochastic nature of many ABMs requires careful consideration of optimization algorithms: even with many simulation runs, some variability in output limits the effectiveness of gradient-based search methods. Stochastic approximation techniques may help, and more heuristic global optimization schemes offer potential as well. The issues of obtaining reliable data from repeated simulation and the effect of spatial scale on resulting dynamics were investigated in Oremland and Laubenbacher (2014a). While that paper presents several techniques for control of ABMs directly via simulation, it would seem more appealing in general to apply the well-developed systems-theoretic tools of optimal control.

## Control Techniques

**Fig. 1:**
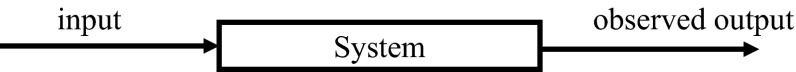
Basic block diagram of a system

The systems-theoretic view is often characterized by input–output relationships, as illustrated in Fig. 1.

The system may constitute a medical patient receiving treatment, with outputs being physiological measures of health. Another example is a spatially distributed ecosystem of pest predators and their prey, with inputs being trapping strategies implemented over time and distributed spatially, and outputs being observations of total pest population. The system may be a society of agents with different incomes and wealth levels, with a control being taxation, as in Sugarscape.

The inputs can be static, like parameters, or dynamic. Dynamic inputs can include control signals designed to influence the system and disturbances, stochastic or deterministic. The observed output signal represents those things we can measure.

A functional relationship between an input control signal *u* and an observed output signal *y* is often referred to as a transfer function:Here, the transfer function *G* is assumed at a minimum to be causal, meaning that the output at any time instant can only depend on the control signal at times up to and including the current time. Other modeling assumptions may include linearity or time invariance.

**Fig. 2:**
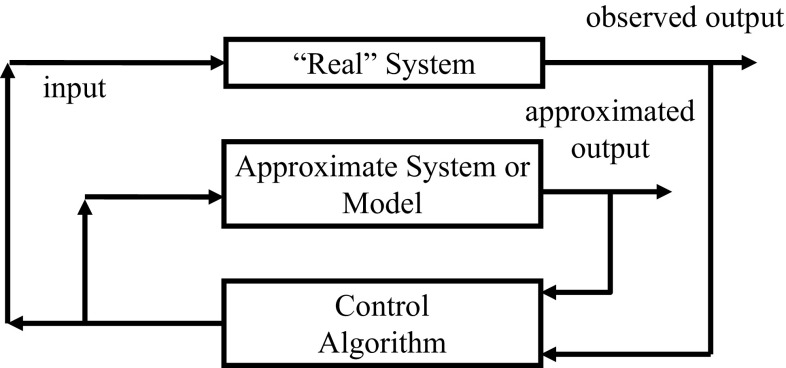
Model-based control block diagram

In many control applications, one builds a model of the system in order to design control signals that will lead to desirable outputs. The model involves two closely related but distinct ingredients: a model that approximates the system’s input/output behavior, and a control algorithm that uses model information to determine appropriate control inputs. Such a circumstance is illustrated in Fig. 2, as we augment the real system of interest with the model and controller:

The possible objectives of control are either to keep a system’s output within some desired operating range of interest, or to move a system from its current state to a more desirable one. Designing controls to meet these objectives is generally approached by choosing an optimization criterion or objective functional to be maximized or minimized.

Often the system is characterized by a dynamic state variable whose value represents the complete state of the system at any given time. To fix ideas, we focus on an optimal control approach, in which we consider the following mathematical formulation:

$$\begin{aligned} {\dot{x}}(t)= & {} f(t, x(t), u(t), \theta )\\ y(t)= & {} h(t, x(t), u(t), \theta )\\ J(u)= & {} {\mathop {\int }\limits _{t_{0}}^{t_{f}}} L(t, x(t), u(t)) \text {d}t + \Phi (x(t_{f})) \end{aligned}$$

in which *x* denotes the state of the system, *u* denotes an exogenous input we refer to as the control, and $\theta$ denotes system parameters such as rate constants, susceptibilities, etc., with the state variable’s rate of change being modeled by the function *f*, which we call simply the dynamics. The second augmenting equation denotes the system’s outputs, quantities we can measure, which we refer to as *y*, with the function *h* modeling the relationship between the state, control, parameter, and output. Finally, the functional*J*,  depending on the control signal *u*,  models our objective for choosing an optimal control.

Among the challenges of modeling, particularly model-based decision and control, is the identification of the state variables needed to capture the dynamics of a complex system with many scales and interacting components. The dynamical system may need to account for spatial heterogeneity (leading often to partial differential equations like diffusions). Other sorts of heterogeneity (e.g., different levels of susceptibility to disease, different levels of metabolism) also make for challenges in state variable modeling approaches. As an example, the effect of simple and straightforward changes in agent movement on the ability to formulate a system-level model (SLM) is presented in Oremland and Laubenbacher (2015). To what extent aggregation can be tolerated in an SLM of a complex heterogeneous system and its associated agent-based model is an important topic, the investigation of which offers exciting mathematical research opportunities.

## Systems Analysis and Control for Agent-Based Models

System-level modeling of an ABM might reasonably be viewed as the same activity as developing a model of the real system represented by the ABM. Indeed, one fruitful view of ABMs is that of experimental surrogate. ABM development often centers very directly on translating the process details of a physical, chemical, biological, or social system into behavioral rules that can be instantiated in code. There are, however, a number of fundamental differences.

First and foremost, the ABM may involve a number of control inputs and parametric settings that are not practically realizable in experimentation. This situation is particularly relevant in biological and social systems, for which experiments may involve difficult ethical questions. Second, the ABM may allow one to monitor processes for which current measurement technologies do not exist. Additional measurements in the computational model can provide important insights into control algorithm performance and stimulate innovation in science and engineering of sensor and other measurement technologies. Third, we note that the simulation can be exercised in a wider variety of configurations and spatial and temporal sampling rates than can most physical, biological or social systems.

**Fig. 3:**
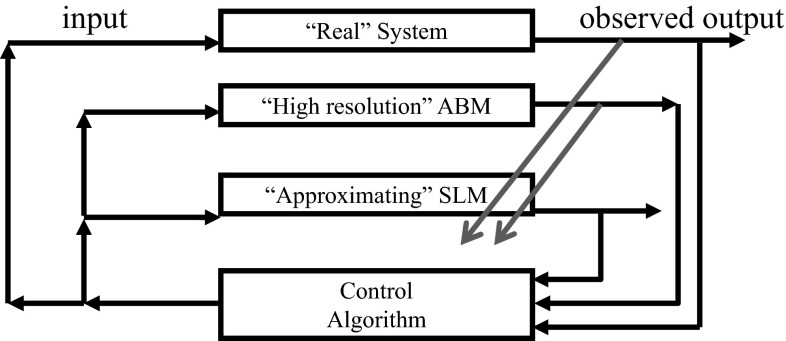
(Color Figure Online) Two levels of modeling in a decision and control loop

Inserting an ABM into the decision and control loop of real systems leads to a slight modification of the control diagram in Fig. 2. The red arrows in Fig. 3 denote the fact that the real system’s output and the ABM’s output may be used to tune the SLM. The manner in which this is done depends on many details of the experiments and the models. Among the more interesting and challenging issues is that inputs and outputs for the real system, the ABM, and the SLM, may be very different data structures.

Consider the first example problem of ecological pest control. The traditional Lotka–Volterra consumer-resource model (like predator-prey) is a pair of ordinary differential equations given by

$$\begin{aligned} {\dot{g}}(t)= & {} \alpha g(t) - \beta g(t)r(t)\\ {\dot{r}}(t)= & {} - \gamma r(t)+\delta g(t) r(t)-u (t)r(t) \end{aligned}$$

in which *g* and *r* denote the total amounts of grass and rabbits as functions of time. The parameters $\alpha$ and $\beta$ denote growth rate and loss rate due to rabbit consumption of the grass, and the parameters $\gamma$ and $\delta$ denote the mortality rate of rabbits and conversion rate of grass energy into newborn rabbits. The function *u*(*t*) gives the harvest rate of the rabbits. While there are many choices of objective functions that will lead to pest reduction, one relatively obvious choice is

$$\begin{aligned} J(u)={\mathop {\int }\limits _{0}^{T}} r(t)+ wu(t)\text {d}t \end{aligned}$$

which we minimize with respect to *u*, subject to the Lotka–Volterra equation constraint. The number *w* represents a weight to balance two costs: in minimizing this functional we attempt to minimizing a combination of the total rabbit population and the harvesting expenses.

In order to project down from the ABM to this model, the total amounts of grass and rabbits can be computed from ABM output. However, the reverse direction is not well-defined: lifting up from the simple Lotka–Volterra model to the state of the ABM is not unique. In particular, the harvesting rate must be distributed as a probability for grass growth across the spatial domain.

This issue presents an important general consideration in developing an SLM for system analysis of an ABM: mapping back and forth between the two state spaces of the models. Generally speaking, the mapping from ABM states to SLM states, which we call a projection, is straightforward. Often the SLM states can be viewed as summary statistics of the ABM, be they total abundances, means, or fractions with specific attributes or properties. Summing the number of agents with a certain property (e.g., all the rabbits or all the grass) produces the necessary projection in many situations.

The mapping from the SLM to the ABM, which we call a lifting, requires more care. ABM states tend to involve much more detail than SLM states, but the summary statistic view can help in lifting: ABM states can often be approximated as random samples from a distribution that depends on a simple parameterization that can be characterized using the SLM state. For example, with a known number of rabbits, we can randomly distribute rabbits across the spatial domain. Lifting the state variable itself is not the only consideration here: lifting the control input, which will be used to influence the ABM dynamics, is also an issue.

We have sketched a very simple approach to one relatively low-complexity example problem by proposing a well-known differential equations model as the surrogate SLM. We note here that there are many approaches to designing the SLM, including (but not limited to) time series models of transfer functions, difference equations (Oremland and Laubenbacher 2014b), ordinary, partial, and stochastic differential equations, polynomial dynamical systems (Hinkelmann et al. 2011), and Markov chains. Within each of these rough categories, many approaches to developing a model exist. One may specify a simple functional form and fit parameters to output. On the other hand, one may create a more mechanistic model, for which the parameters have direct conversions to and from the ABM parameters. In either case, one must still perform a parameter estimation of some sort. With the fitted model in hand, one must then compute the optimal control. Table 1 delineates potential modeling approaches, parameter estimation methods, and control design techniques.

**Table 1:** A partial break-down of modeling, estimation, and control methods

Modeling approaches	Parameter estimation methods	Control design techniques
1. Discrete input–output	Output least squares 1,4,5	Pontryagin’s maximum principle 1,4,5
2. Markov chain	Equation error 4,5	Dynamic programming 1–5
3. Polynomial dynamical systems	Maximum likelihood 1–5	Large scale constrained optimization 1–5
4. Difference equations	Maximum a posteriori 1–5	
5. Differential equations		

To illustrate some ways in which a system-level problem can be investigated using an ABM, we return to our rabbits-and-grass model described above. A relatively simple problem, the rabbits-and-grass simulation nonetheless exhibits many of the key behaviors of ABMs. We consider here three distinct routes to system-level analysis.

1. Stochastic optimization applied directly to the ABM;
2. Mechanistically derived SLM constructed as a surrogate for the ABM; and
3. Empirically fit SLM constructed as a surrogate for the ABM.

### Stochastic Optimization Applied Directly to the ABM

Here we view the ABM as an input–output system, much like an experiment with settings and measurements. We consider the homogeneous rabbits-and-grass model, and we apply a harvesting strategy defined as follows. For a full description, see Oremland and Laubenbacher (2014a). At each ABM time step, harvesting may or may not be applied. The harvesting has an effectiveness parameter, modeled as a probability. The probability creates a stochastic mortality for each rabbit. The harvesting remains in effect for a second time step, but its effectiveness is reduced by half. The control is a binary time sequence of apply/do not apply, uniformly distributed across the region.

The objective in this example has two features: minimize a weighted combination of the rabbit population and the level of harvesting. We consider a Pareto approach that allows us to trade off these two objectives, implementing the optimization with a genetic algorithm. In this approach, we construct a set of “seed” controls, which, again, is a set of binary sequences representing the harvesting schedule. Each of these is evaluated in the ABM, with the number of surviving rabbits as output. We estimate the Pareto frontier by removing all controls whose objective pair (surviving rabbits, days of harvesting) is dominated by any other control. Controls that persist are paired to produce offspring by randomly choosing parent sequence components. The details of the procedure can be found in Oremland and Laubenbacher (2014a).

**Fig. 4:**
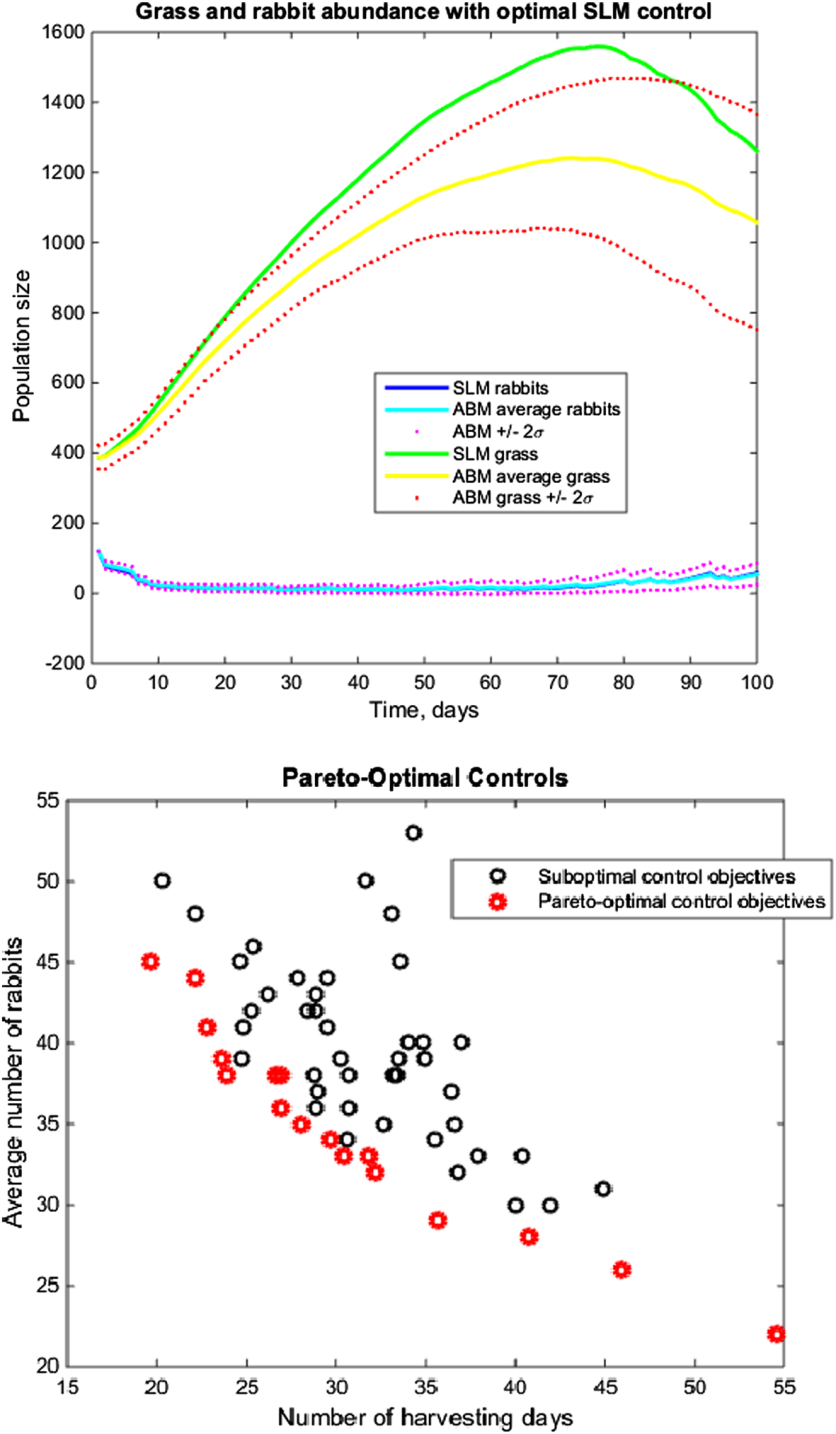
(Color Figure Online) *Upper panels* show the time course of the SLM and ABM, the latter with an average of 100 realizations and +/- 2 standard deviations. The *top* shows the system with no control applied, and the *bottom* illustrates a Pareto-optimal control. *Third panel* shows average number of rabbits versus days of harvesting. The *red symbols* represent Pareto points, while the *black symbols* show suboptimal controls

It should be noted that generating the seed controls is a difficult problem in and of itself. Even in this approach of applying a heuristic optimization scheme, we need an approximating SLM to aid in control construction. A discrete dynamical system for the rabbits and grass is used in conjunction with the genetic algorithm to develop seed controls. The difference equation takes the form

$$\begin{aligned} r(t+1)= & {} ar (t)+br(t)g(t)\\ g(t+1)= & {} g(t)+\gamma (1-g(t))-{\frac{r(t)g(t)}{N}}, \end{aligned}$$

in which the parameters *a*, *b*, and $\gamma$ are estimated by least squares comparison with ABM simulation output, and *N* denotes the number of grid cells in the ABM. In Fig. 4 we illustrate the matching of the SLM to the ABM in the case of no control applied. The resulting Pareto frontier is also shown, yielding the number of rabbits remaining at the final time as a function of the control effort, as modeled by the number of days harvesting is applied.

This approach provides a suite of solutions, each of which may be optimal, depending on the preferences of the modeler. Hence, the optimal solution chosen can be thought of as a ‘managerial’ decision. While this approach demonstrates a means by which optimal control results can be obtained directly from ABM execution, it lacks a level of mathematical rigor that may be necessary for conclusive statements.

### Mechanistic SLM Derived for ABM Approximation

In this approach, we also view the ABM as an input–output experimental system. We consider the basic processes captured in the ABM, with an eye toward developing a model amenable to control approaches. We construct a simple consumer-resource ODE system with control. For a full description, see Federico et al. (2013).

The model we use is of the form

$$\begin{aligned} {\dot{g}}(t)= & {} a_{1} (a_{2}-g(t))-f (g(t)) r(t)\\ {\dot{r}}(t)= & {} b_{1}f (g(t)) r(t)- b_{2}r(t)-u(t)r(t) \end{aligned}$$

in which

$$\begin{aligned} f(x)={\frac{a_{1}a_{3}x}{1+a_{3}a_{4}x}}, \end{aligned}$$

and *u*(*t*) denotes the control, which is a harvesting rate between 0 and 1, representing the fraction of rabbits harvested per unit time. Thus, the grass grows according to a logistic model in the absence of rabbits. The rabbits convert grass to rabbit offspring according to a type of Monod kinetics, and the rabbits have a simple linear mortality rate. The (time dependent) control increases that mortality rate. Of the six parameters in the model, $a_{1}$ and $a_{2}$ are directly computable from the ABM parameters, while the other four, $a_{3},a_{4},b_{1}b_{2}$ must be inferred from fitting the SLM to SBM output.

The control objective is specified as minimizing the functional

$$\begin{aligned} J(u)={\mathop {\int }\limits _{0}^{T}} r(t)+c_{1} u(t)+c_{2} u^{2}(t) \text {d}t \end{aligned}$$

in which the parameters $c_{1}$ and $c_{2}$ are tunable in order to achieve desirable qualitative behavior. Without $c_{2}$, we can view the parameter $c_{1}$ as balancing the two objectives of the Pareto specification in the previous control methodology. The parameter $c_{2}$ plays a somewhat technical mathematical role (as is discussed in Federico et al. 2013) for improving the performance of the numerical solution approach.

The solution approach of Federico et al. (2013) is closely related to Pontryagin’s principle: a forward-in-time state equation is supplemented with a backward-in-time co-state equation, the latter being a characterization of Lagrange multipliers in this constrained optimization problem. The equations are solved iteratively, and the converged pair produces the optimal control. This problem, solved by itself without the context of the ABM, leads to a time schedule of harvesting rates.

To lift the resulting control to the ABM, just like the harvesting strategy of the “brute force” optimization above, this rate is used as a random mortality, applied uniformly across the domain. Each rabbit will be harvested with probability *u*(*t*).

**Fig. 5:**
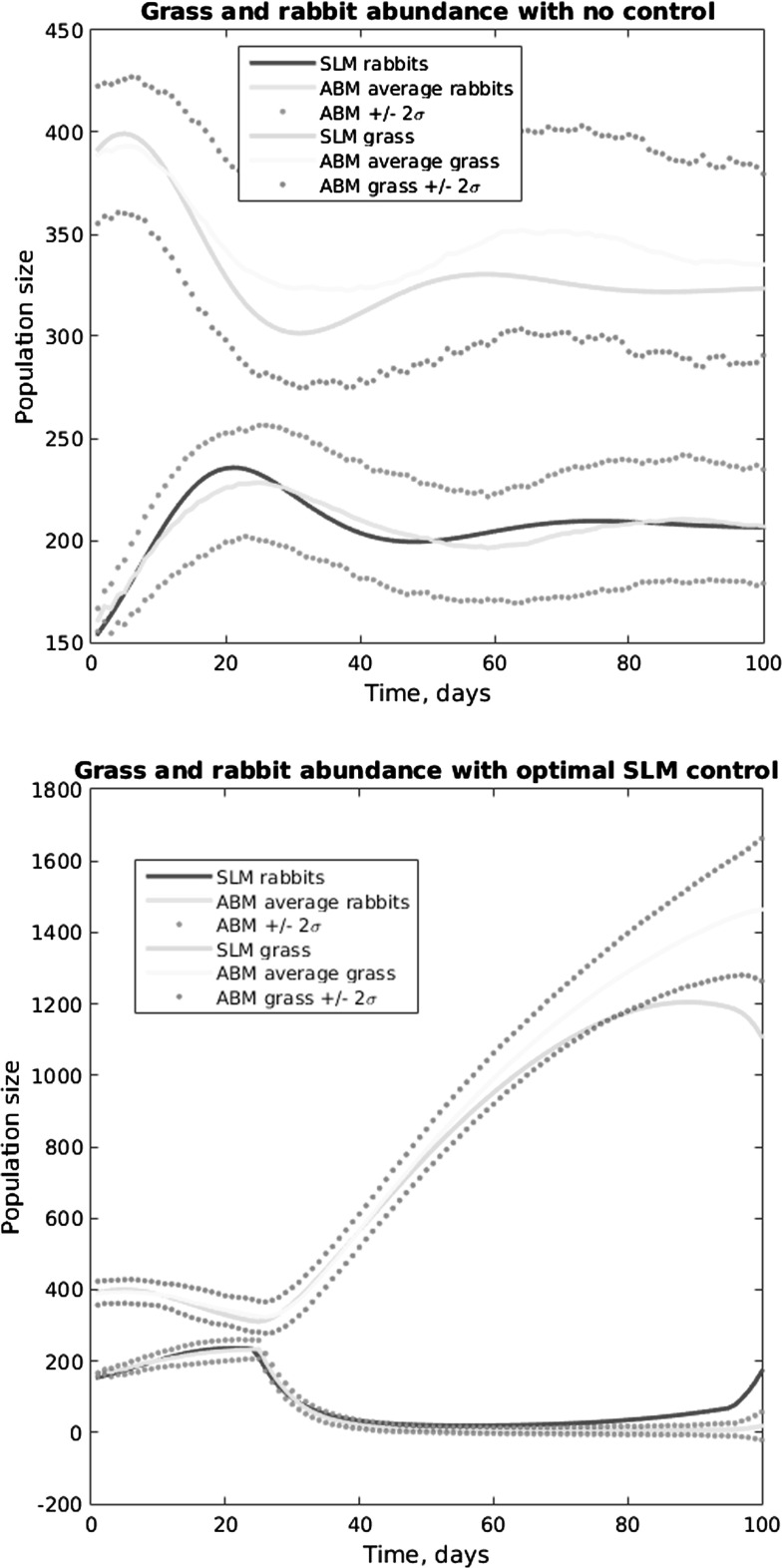
(Color Figure Online) Time course of grass and rabbit abundance, comparing ABM and SLM. *Upper panel* compares the models without any control applied, while the *lower panel* compares the two with the SLM’s optimal control

The work in Federico et al. (2013) contains a number of explorations of this approach, including a heterogeneous grass growth environment. In that heterogeneous environment case, the optimal controls coming from the aggregate differential equation system did not work well in the ABM. In Fig. 5, we illustrate the approach’s results within a simple homogenous environment. The figure shows the time course resulting from the application of the consumer-resource -based optimal control to the ABM. In the upper panel, we see the model’s fit to the ABM data when no control is applied. The lower panel shows the time course when the optimal control is applied, showing a highly reduced number of rabbits. In distinction from the previous approach, the cost functional in this situation contains a fixed (if implicit) balancing of the “tolerable” levels of control effort and residual pest population. The Pareto frontier in the previous approach would amount in this case to a parametric plot of the control effort and residual rabbit population as a function of the parameters $c_{1}$ and $c_{2}$.

### Empirical SLM Derived for ABM Approximation

Here, we again view the ABM as an input–output experimental system. We select the output statistics to control: namely the abundance of rabbits. Our goal, once again, is to devise a harvesting strategy, uniformly applied across the domain, to reduce the abundance of the pest rabbit population.

Rather than consider the mechanistic approach of modeling the rabbit/grass dynamics as consumer-resource, we simply consider the most desirable state of the system, which is every cell populated by grass with no rabbits present. To achieve this state while controlling the level of harvesting effort is the objective. Toward that end, we consider a rabbits-and-grass model with an initial state of $(r_{0}, g_{0})=(0,N)$ and the desired final state of $(r_{f}, g_{f})$, where *N* denotes the number of cells in the ABM. We note that this desired final state is an equilibrium: if there are no rabbits to begin with, the grass will eventually populate every cell. It is unstable; however, as soon as a single rabbit is introduced, the population can and will increase according to the rules of the ABM.

We model the system as a simple linear system, which we envision as being linearized around the desired final state:

$$\begin{aligned} {\dot{x}}=A(x-x_{f})+Bu, \end{aligned}$$

in which *x* denotes the state pair $x=(r,g)^{T}$, $x_{f}=(r_{f},g_{f})^{T}=(0,N)^{T}$, *A* is a $2\times 2$ matrix, and *B* is a $2\times 1$ matrix, both of which must be inferred from the ABM. Assuming that the harvesting does not directly lead to grass abundance change, one may take $B=(b,0)^{T}$ with one unknown parameter. The model here is not necessarily expected to fit the dynamics over the whole range of possible rabbit and grass states; rather, it is meant as a means to the end of constructing a feedback control gain.

The fitting approach is as follows. The differential equation can be approximated by an Euler step, for short time *h*, as

$$\begin{aligned} x(h)-z=A(z-x_{f})+Bv, \end{aligned}$$

in which *z* and *v* denote initial state and control values. By selecting a random sample of initial states and control values, say $(z_{i}, u_{i}), i=1,2,\ldots ,n,$ and by evaluating the ABM for a short time at these values, we obtain an “experimental sample,” which we denote by $Y_{i}(h)$. The values of *A* and *b* may then be obtained via linear regression. It is important to note that this technique depends on very short time executions of the ABM, as opposed to the parametric fits of the previous approach that rely on long time simulations. The approach we describe here is closely related to Kevrekides’ so-called “equation free” analysis technique (Kevrekedis et al. 2004; Armaou 2004). The application of this technique to the rabbits-and-grass ABM is described in detail in Fitzpatrick (2016b).

Control strategies can be derived in a number of ways. In particular, one may apply the forward-backward iteration of Federico et al. (2013) mentioned above. A different, much simpler approach, is the linear quadratic regulator. We denote by *w* the state perturbation from equilibrium: $w(x-x_{f},)$ so that ${\dot{w}}=Aw+Bu$, and we define the cost functional

$$\begin{aligned} J(u)={\mathop {\int }\limits _{0}^{\infty }}w^{T} Qw+u^{T} Ru \,\mathrm{d}t \end{aligned}$$

in which *Q* is a $2 \times 2$ nonnegative definite matrix, and *R* is a positive scalar. As with the constants $c_{1}$ and $c_{2}$ in the objective of the mechanistic SLM approach, these matrices contain tuning parameters used to achieve desired results. For this problem, we choose $$Q= \left[ \begin{array}{ll} 1 &{} 0 \\ 0 &{} 0 \\ \end{array} \right] $$, so that the objective becomes

$$\begin{aligned} J(u)={\mathop {\int }\limits _{0}^{\infty }}r^{2}(t)+Ru^{2} (t) \text {d}t \end{aligned}$$

similar to the mechanistic SLM objective. The major advantage to this linear–quadratic formulation is that the control is determined by the solution of an algebraic Riccati equation:

$$\begin{aligned} u= & {} -Kx, \hbox { with }\\ K= & {} R^{-1} B'P, \hbox { where}\\ 0= & {} A'P+PA+Q-PBR^{-1} B'P \end{aligned}$$

**Fig. 6:**
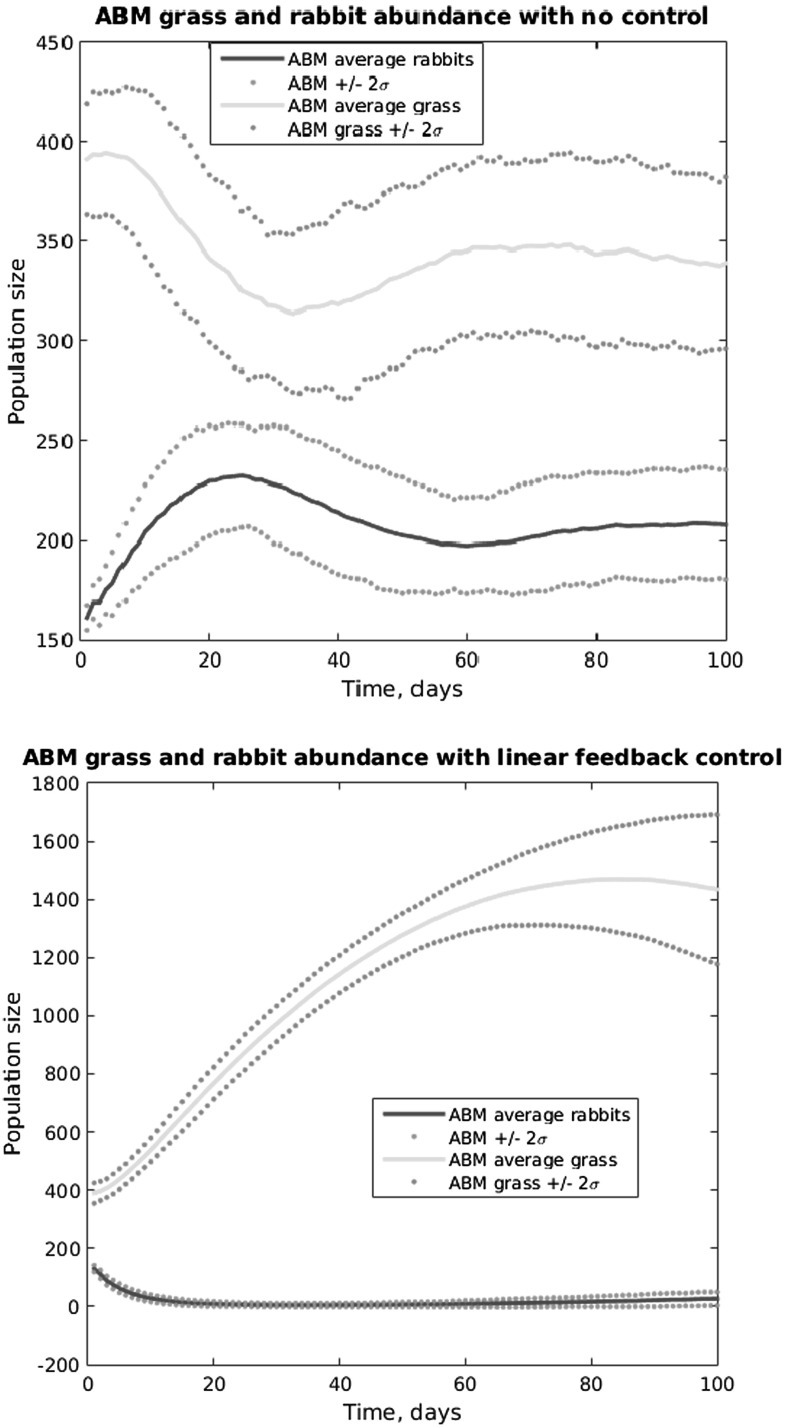
(Color Figure Online) Time course of grass and rabbit abundance. *Upper panel* compares the models without any control applied, while the *lower panel* compares the two with the SLM’s optimal control

So the solution of this final equation, a quadratic equation for a symmetric $2 \times 2$ positive definite matrix, leads directly to a simple formula for the control as a constant gain matrix *K* applied to the rabbit and grass state. Lifting the control is again accomplished by harvesting with probability *u*(*t*) across the region of interest. In analogy with Fig. 5 for the mechanistic nonlinear SLM, Fig. 6 shows the results of the ABM with no control applied and with the optimal linear–quadratic SLM control applied.

## Discussion

ABMs offer an interesting investigative tool for science, engineering, public health, and public policy. Replicating important features of real-world systems with relatively simple rule-based computational models make ABMs attractive alternatives to traditional mathematical models. Using ABMs as decision tools to improve or optimize real-world systems, however, remain a significant challenge. In this “Perspectives” article, we have examined a number of systems-level control approaches that might be applied to ABMs, using the simple Rabbits–Grass–Weed ABM as a pest control example.

In approaching an ABM from the point of view of control, a number of key problems arise, especially when SLM approximations are used. Most fundamental is the mapping of inputs and outputs between ABM-scale and SLM-scale quantities, which we have called “lifting” and “projection.” Next, one must consider the necessary complexity of the SLM required to capture the ABM dynamics relevant to the control inputs and objective.

While the simple example of the Rabbits–Grass–Weed pest control problem allows us to illustrate some features of ABMs and suggest some approaches to their control, many challenges remain for a more general toolset for the system-level analysis of ABMs and their control. To develop mathematical approaches, we must pay careful attention to a number of issues, including (but by no means limited to)

- Methods for dimension reduction;
- The extensive heterogeneity possible for individual agents;
- Agent adaptation;
- Multiple time scales that emerge in complex ABMs;
- Taxonomy of ABMs with respect to different SLM paradigms and success of one of the three approaches outlined in this paper (or others);
- Rigorous methods for projection and lifting in order to map between analytical and simulation models;
- Efficient computational experimental design; and
- Uncertainty quantification in assessing SLM–ABM compatibility.

Ronald Fisher proposed a statistical framework (Fisher 1926) that defined quantitative experimental design, and Wald’s seminal work (Wald 1939) energized research in statistical decision theory. These and related works provide solid theoretical foundations and practical techniques for designing experiments and optimizing outcomes based on statistical assumptions about the underlying experimental processes. As simulation models have come to complement physical and biological experimentation, statistical design and decision theory has been revisited (see e.g., Morris et al. 1993), and progress has been made in leveraging some of the unique properties of ABM models for comparison with experiment (Grimm and Railsback 2005; Grimm et al. 2005; Thorngate and Edmonds 2013). However, the structure of simulation models often contains a great deal of information, much of which yields analytical leverage for design and control. The opportunities are great for developing mathematical and statistical techniques for a new experimental design and decision theory for complex simulations such as ABMs. They bring a powerful modeling technology to scientists, enabling investigations that had previously been prevented from using models because of mathematical barriers-to-entry. For our purposes, we made the assumption that the ABM is a faithful representation of the actual biological system of interest, so that the goal is to develop control strategies for the ABM, with the implicit understanding that their efficacy for the real system will depend on the quality of the ABM. The main argument we present in this paper is that for the purpose of specific control and optimization problems, a given ABM can beneficially be viewed as a surrogate for the real system of interest that can be used for the construction of equation-based models for which control approaches are available. These equation-based models need to be faithful to the ABM and, by extension, the biological system of interest, ONLY to the extent that they capture correctly those aspects of the ABM that pertain to the specific control problem.

We have presented some possible ways to begin accomplishing this, even though the work to date is ad hoc and does not address many of the obvious questions that remain unanswered. Our investigations have mostly focused on two models, Sugarscape and Rabbits-and-Grass, in some variations. They were chosen because they include features found in many ABMs in the literature, but they are arguably too simple to draw conclusions about the broader applicability of our methods. Furthermore, the methods themselves are ad hoc and have not been analyzed rigorously. Are there common principles in how an ABM is approximated by a difference equations model or a PDE model? What are the limits of dimension reduction methods, and are there other comparisons of models that should be used to judge the faithfulness of a reduced model? Answers to these and many other questions await further research, and an important motivation for this paper is the hope that it will engage others in work on these problems. One important tool would be a suite of benchmark ABMs, representative of the different ABM types in use, that illustrate the applicability and limitations of the various techniques. And, of course, other, complementary, techniques will need to be developed.
